# Improved phase-to-height mapping method combine with device attitude

**DOI:** 10.1038/s41598-024-59102-7

**Published:** 2024-04-26

**Authors:** Shuhuan Han, Yanxi Yang, Xinjie Li, Xubo Zhao, Xinyu Zhang

**Affiliations:** 1 School of Automation and Information, Xi’an University of Technology, Xi’an, 710048 China; 2 Shaanxi Key Laboratory of Complex System Control and Intelligent Information Processing, Xi’an, 710048 China

**Keywords:** Electrical and electronic engineering, Applied optics, Optical techniques

## Abstract

Phase-to-height mapping is one of the important processes in three dimensional phase measurement profilometry. But, in traditional phase-to-height mapping method, the measurement accuracy is affected by device attitude, so it needs saving a large amount of mapping equations to achieve high-quality phase-to-height mapping. In order to improve that, this paper proposes an improved phase-to-height mapping method combine with device attitude. Firstly, we get the unwrapped phase of the target. Then, using generalized regression neural network is used to reduce the offset of phase information at the same height due to the randomness of device attitude. Last, the phase-to-height mapping is completed by substituting the unwrapped phase (the difference between having detected object and no detected object) of eliminate the offset into improved phase-to-height mapping method. Experimental results show that the proposed method could achieve high-quality phase-to-height mapping with less mapping equation and less memory space. Compared with the nonlinear phase-to-height mapping method (probabilistic neural network to eliminate phase offset), its accuracy is improved by 44.30%. Compared with the nonlinear phase-to-height mapping method (radial basis function neural network to eliminate phase offset), the accuracy is improved by 39.58%.

## Introduction

Phase measurement profilometry (PMP) system consisting of projector and camera has the advantages of simple structure, strong anti-noise interference ability and full-field measurement^1–5^, and has been widely used in biomedical, industrial measurement, entertainment industry and other fields^6–8^. The principle of this system to achieve three-dimensional measurement is that the unwrapped phase corresponding to the height in the field of view could be obtained by analyzing the captured fringe images, and then the real height of the detected object could be recovered from the unwrapped phase through the calibrated phase-to-height mapping method. In order to obtain the available height information in practice, phase-to-height mapping is necessary, and the accuracy of the phase-to-height mapping is directly related to the final measurement accuracy, so how to achieve accurate mapping simply and effectively is one of the research objectives in this field. Takeda et al.^9^ first propose a simple explicit phase-to-height mapping method, which is solved by linearly mapping the measured height to the phase difference. However, in real life, this method has certain installation limitations, and the relationship between the measured height and the phase difference is a complex nonlinear relationship, so this model is difficult to realize in practice. Huang et al.^10^ propose an improved explicit phase-to-height mapping method, which does not require too many installation limitations and has stronger applicability. However, this method requires the attitude analysis of camera and projector in advance, which makes the implementation process of this method relatively complicated, so it is not only difficult to implement, but also easy to introduce errors. Zhou et al.^11^ propose a direct phase-to-height mapping method with an inverse linear relationship between phase and height. However, due to the influence of various factors, phase and height cannot fully satisfy the above inverse linear phase-to-height mapping relationship in practice. Based on the above inverse linear phase-to-height mapping method, Li et al.^12^ and Jiang et al.^13^ add a quadratic term of unwrapped phase to make the model become a nonlinear model to correct the influence of errors in the actual situation. Although the above two methods do not require a complex process of fitting system parameters and could obtain the implicit parameters through several phase information corresponding known heights, in the actual situation, a mapping equation needs to be obtained at each pixel position, which makes this method need to occupy a large amount of memory space to store the above mapping equations. The application of this method is greatly limited.

Therefore, in order to solve the above problems, this paper proposes an improved phase-to-height mapping method combine with device attitude. The method takes into account the influence of the attitude of digital light procession (DLP) projector and industrial camera on the unwrapped phase (the difference between having detected object and no detected object) in the PMP system, and uses generalized regression neural network (GRNN) to eliminate the influence of the unwrapped phase offset caused by the above reasons, so that the influence on the unwrapped phase at the same height is greatly reduced. Then, we substitute the unwrapped phase obtained before into the proposed method to achieve high quality phase-to-height mapping.

### Error analysis

Nowadays, the commonly used phase-to-height mapping methods include: explicit phase-to-height mapping method and implicit phase-to-height mapping method. The explicit phase-to-height mapping method involves many calibration parameters, and the calibration process is relatively complicated, so it is not only complex to implement, but also easy to introduce additional errors. The commonly used equation of the implicit phase-to-height mapping method is nonlinear phase-to-height mapping method, as shown in Eq. (1)^14–17^, Where, $\alpha (x,y)$, $\beta (x,y)$ and $\gamma (x,y)$ are the three fitting parameters.

1 $$ \frac{1}{h(x,y)} = \alpha (x,y) + \frac{\beta (x,y)}{{\psi (x,y)}} + \frac{\gamma (x,y)}{{\left[ {\psi (x,y)} \right]^{2} }} $$

The above nonlinear phase-to-height mapping method does not need to obtain the internal and external parameters of the system, and includes the distortion and nonlinearity of the system in the parameter fitting process, and directly uses several unwrapped phase of known heights to fit the relationship between phase and height. However, there are still some problems, which are analyzed as follows.

**Figure 1:**
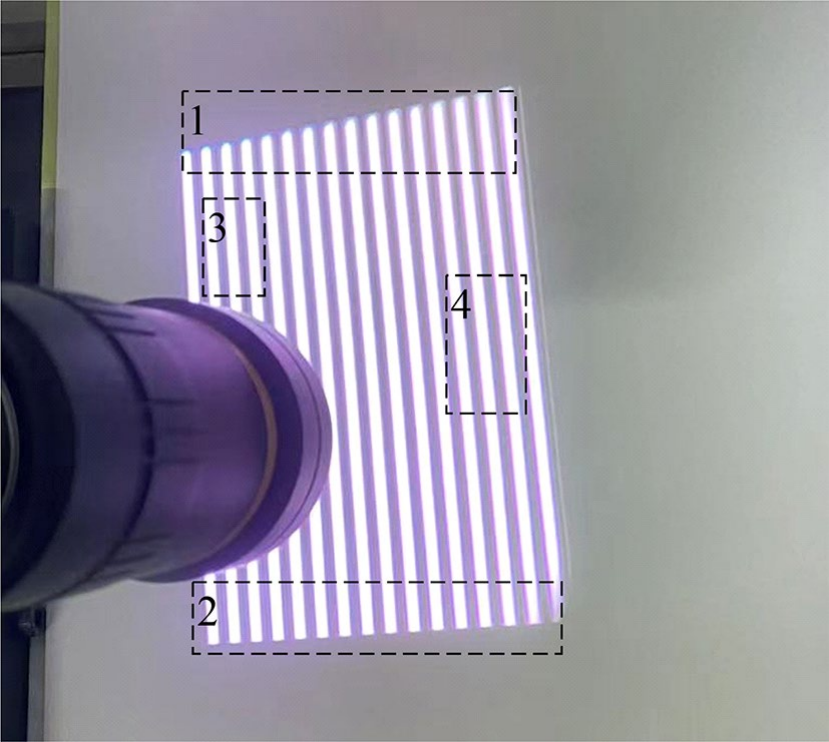
Projected fringe image in the actual environment.

**Figure 2:**
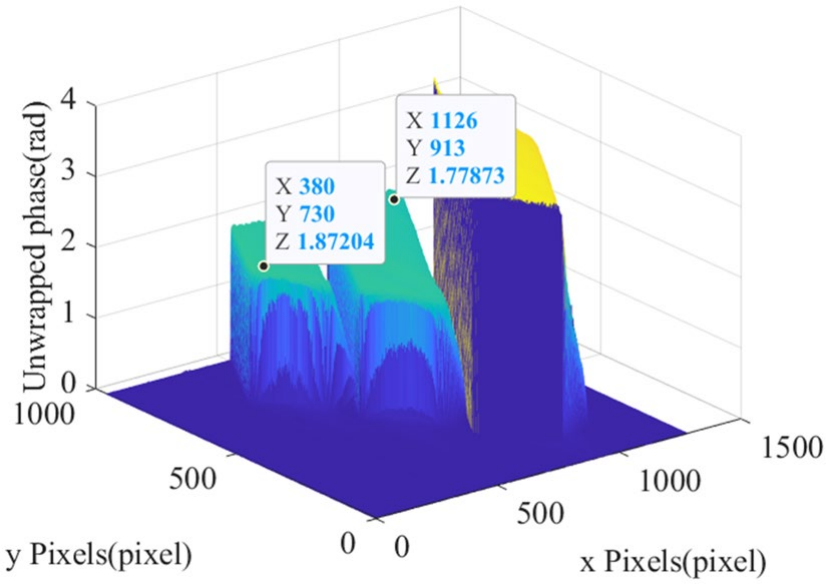
Unwrapped phase of different heights gauge blocks.

Figure 1 shows the projected fringe image in the actual environment. By observing and comparing region 1 and region 2 in Fig. 1, it could be seen that the edge of the projected fringe image is obviously distorted. By observing and comparing region 3 and region 4 in Fig. 1, it could be seen that the width of the fringe in the projected image at different positions has changed. It could be seen from the analysis that the installation attitude of DLP projector causes a certain offset in the corresponding unwrapped phase of the target of the same height, as shown in Fig. 2:

Figure 2 shows the unwrapped phase of different heights gauge blocks. The height of gauge blocks is 15 mm, 15 mm and 25 mm from left to right. Since the output height $h(x,y)$ of Eq. (1) is only related to the unwrapped phase $\psi (x,y)$, if the phase-to-height mapping of all pixel positions adopts only one or several correlation mapping equations, this scheme is difficult to achieve in principle due to the influence of the above unwrapped phase offset. If a correlation mapping equation is obtained for each pixel position, the correlation mapping equation cannot be generalized, and a large amount of memory space is required to store the mapping equation, which greatly limits the practicability of this method. Therefore, it is very important to propose a method that could consider the device attitude and achieve high quality phase-to-height mapping with fewer mapping equations.

### The proposed method

**Figure 3:**
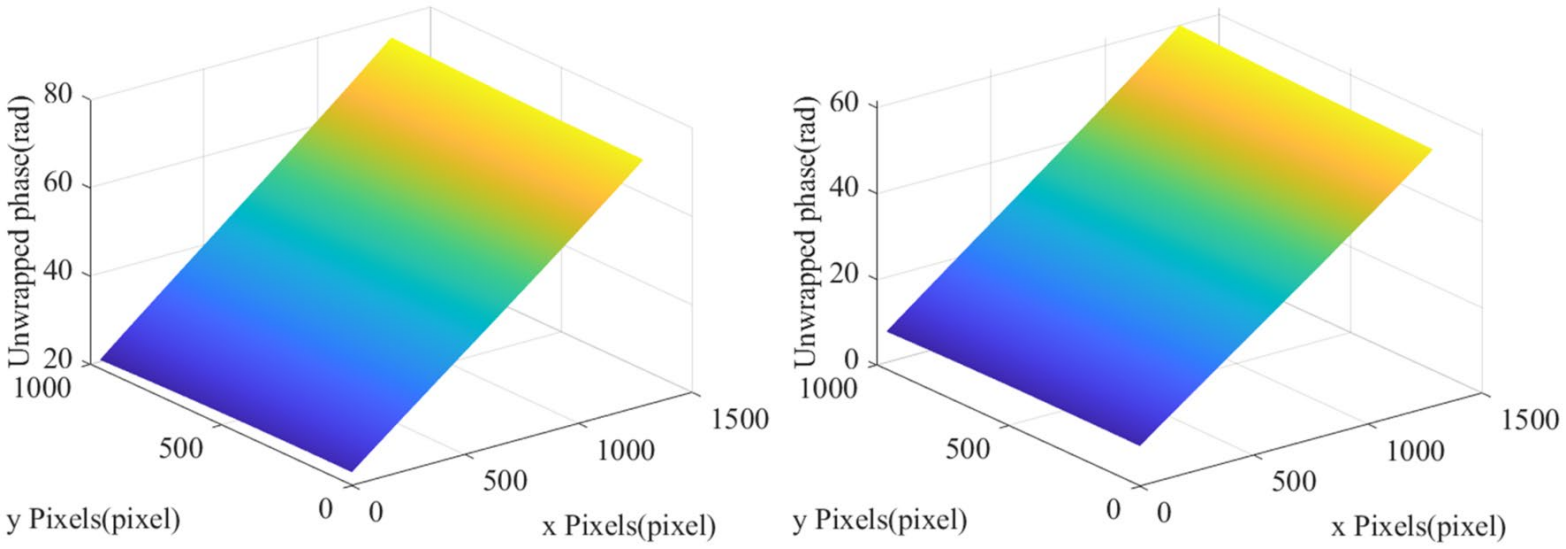
Unwrapped phase of different measured heights.

**Figure 4:**
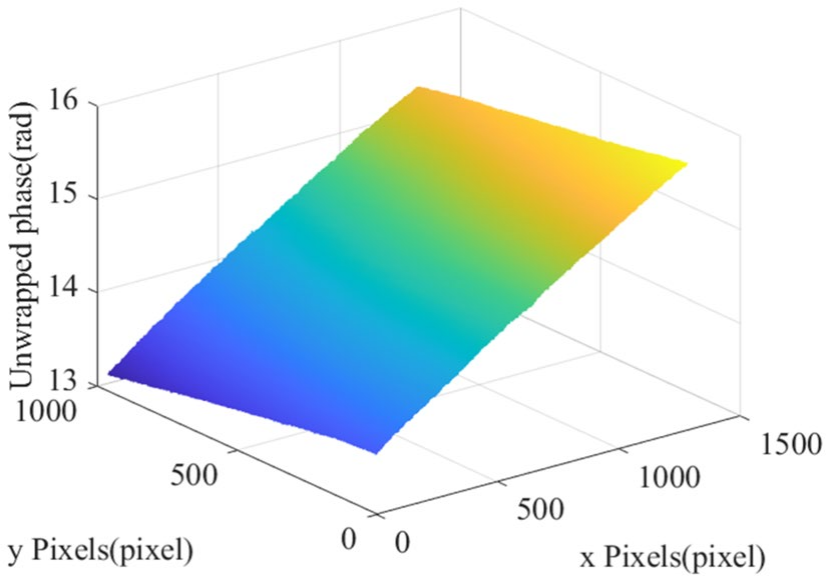
Phase difference effect diagram.

In order to obtain a new high-quality phase-to-height mapping method, we carry out the following analysis: First, the unwrapped phases of reference plane in different measured heights (the measured height is the distance between the camera or the DLP projector and the measured surface) are respectively got and denote as $\psi_{1}^{unwrap}$ and $\psi_{2}^{unwrap}$. The effect diagram is shown in Fig. 3:

Then, we substitute $\psi_{1}^{unwrap}$ and $\psi_{2}^{unwrap}$ into Eq. (2) to obtain the phase difference of the two unwrapped phases, denote as $\psi_{diff}^{unwrap}$, where: $\psi_{{}}^{thre}$ is the threshold of phase error judgment, and its effect is shown in Fig. 4.

2 $$ \psi_{diff}^{unwrap} = \left\{ {\begin{array}{*{20}l} {0,\psi_{1}^{unwrap} - \psi_{2}^{unwrap} > \psi_{{}}^{thre} } \hfill \\ {0,\psi_{1}^{unwrap} - \psi_{2}^{unwrap} < 0} \hfill \\ {\psi_{1}^{unwrap} - \psi_{2}^{unwrap} ,else} \hfill \\ \end{array} } \right. $$

Based on the analysis of Fig. 4, it could be seen that due to the influence of the attitude of DLP projectors, industrial cameras and other device, the unwrapped phase corresponding to the same height have a certain offset, and the unwrapped phase offset has a certain correlation with its coordinate and GRNN is a kind of local approximation neural network, which has the advantages of strong nonlinear mapping ability, good robustness and flexible network structure, so it is often used to solve nonlinear problems^18,19^. In view of this, we introduce GRNN to eliminate the influence of device attitude on phase information, so that the phase offset in the unwrapped phase corresponding to the same height is greatly reduced:

- *Step 1* The coordinates $(x,y)$ of the unwrapped phase offset are taken as inputs and the offset of unwrapped phase at corresponding coordinates are taken as outputs to train the GRNN model, as shown in Eq. (3):
  3 $$ \psi_{offset}^{unwrap} = GRNN(x,y) $$
- *Step 2* Let $\psi_{diff}^{unwrap}$ subtract $\psi_{offset}^{unwrap}$ to get the phase difference of the unwrapped phase that eliminates the effect of the unwrapped phase offset, denote as $\psi_{diff}^{\prime unwrap}$, as shown in Eq. (4) :
  4 $$ \psi_{diff}^{\prime unwrap} = \psi_{diff}^{unwrap} - \psi_{offset}^{unwrap} $$
- *Step 3* Substitute $\psi_{diff}^{\prime unwrap}$ as input into the phase-to-height implicit mapping method to obtain high-quality phase-to-height mapping results.

Summarizing the above, as shown in Eq. (5) :

5 $$ \frac{1}{h(x,y)} = \alpha + \frac{\beta }{{\psi_{diff}^{unwrap} (x,y) - GRNN(x,y)}} + \frac{\gamma }{{\left[ {\psi_{diff}^{unwrap} (x,y) - GRNN(x,y)} \right]^{2} }} $$

Based on the above analysis, this paper proposes an improved phase-to-height mapping method combine with device attitude, the pseudo-code is shown as follows:

**Algorithm:**
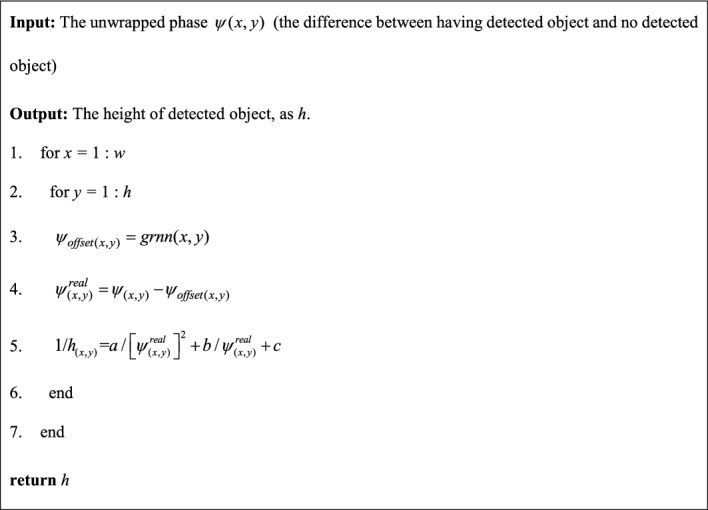
Improved phase-to-height mapping method.

## Experiment

**Figure 5:**
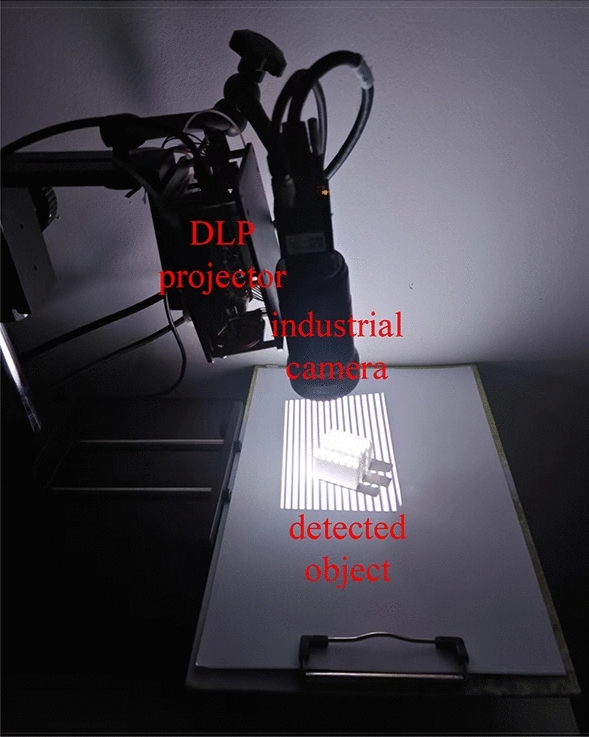
Phase measurement profilometry system.

In order to verify the effectiveness of the proposed method, we set up a PMP system, which is composed of Microvision MV-EM120C industrial camera (resolution 960 pixel × 1280 pixel × 3 pixel) and DLP4500 projector^20–24^, as shown in Fig. 5.

In this experiment, we use a projector to project fringe images onto several sets of white ceramic gauge blocks of different heights (15 mm, 25 mm, 30 mm and 40 mm) on the reference plane and the reference plane. Then, the fringe images capturing by industrial cameras are analyzed to obtain the unwrapped phase of the reference plane and the unwrapped phase modulated by the white ceramic gauge blocks of different heights. Next, the unwrapped phases containing the height information of the gauge blocks subtract the unwrapped phase of the reference plane to obtain the phase difference. Finally, the measurement results are calculated according to the proposed method.

In order to verify the superiority of the proposed method, we set up a comparison experiment to compare with the proposed method, and we choose the following methods to conduct the comparison experiment: direct phase-to-height mapping method (reference^11^, denote as method 1), nonlinear phase-to-height mapping method (reference^12,13^, denote as method 2), phase-to-height mapping method (probabilistic neural network (PNN) to eliminate phase offset, denote as method 3), and phase-to-height mapping method (radial basis function (RBF) neural network to eliminate phase offset, denote as method 4). We record the size of the memory space occupied by mapping equations about different mapping methods (sufficient training samples, the amount is limited only by computer memory), as shown in Table 1, and abandon fitting of the corresponding coordinate when the phase or height is infinite.

**Table 1:** The size of the memory space occupied by mapping equations about different mapping methods.

Method	Occupied memory space size (MB)
Method 1	1480
Method 2	1480
Method 3	3.22
Method 4	0.28
The proposed method	17.1

It could be seen from Table 1 that the memory space required for method 1 and method 2 is relatively large and cannot be used in many cases. For better comparison, we make method1(method 2) only have a mapping equation for the measurement system in this paper. In this case, method 1(method 2) is denoted as method 1–2 (method 2–2).

**Figure 6:**
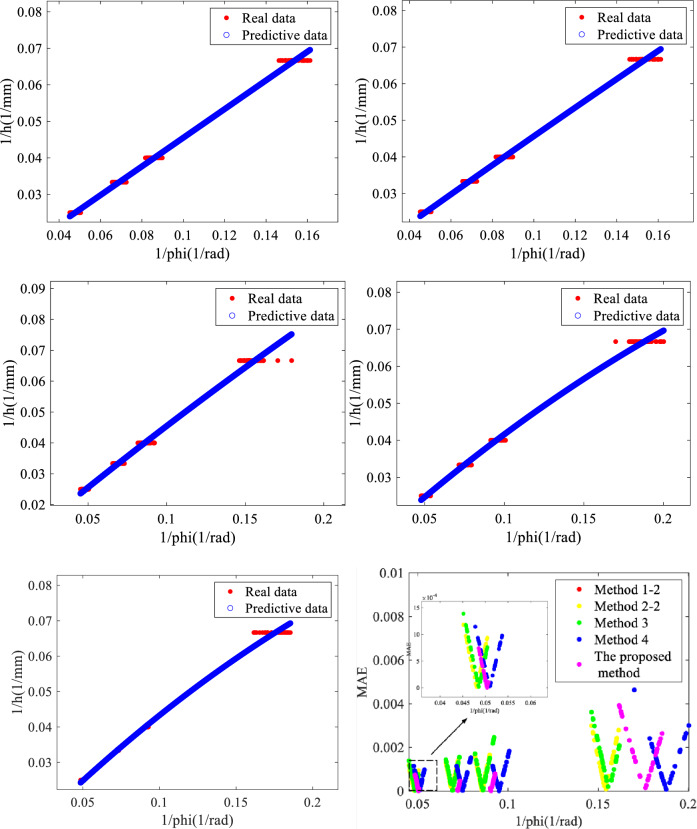
Comparison result of different methods (1) Method 1–2 (2) Method 2–2 (3) Method 3 (4) Method 4 (5) The proposed method (6) The mean absolute error for each method.

Then we randomly select 150 phase-to-height conversion results of the same coordinates among all the results of different methods and compared them with their real height, as shown in Fig. 6.

In Fig. 6, ‘Real data’ are the phases to the real height measured objects by using hardware equipment and ‘Predictive data’ are the height information obtained through phase mapping using the proposed method. As could be seen from above, the proposed method could get height information corresponding to all phases within the defined domain. So it has great practicality and the error fluctuation of the height obtained by the proposed method is smaller than other comparison methods. Therefore, it is concluded from the above experimental analysis that the proposed method could achieve high-quality phase-to-height mapping. In order to more accurately analyze the performance and accuracy of the proposed method, we randomly selected different height gauge blocks, and then calculated the root mean square error (RMSE)^25–27^ between the real height and the predictive height by different methods of the gauge block in some of its regions, we substitute the phase of ‘Real data’ into mapping equations, obtaining the data related to ‘Real data’ in ‘Predictive data’ and record it in Table 2. Where: The classification mechanism in this paper is as follows: The total number of each group data is guaranteed to be 150 groups, and the data that does not meet the region threshold is defined as zero.

**Table 2:** RMSE between the predictive height obtained by different methods and the real height.

Method	1/h			
	$\left[ {{0},{0}{\text{.03}}} \right]$	$({0}{\text{.03,0}}{.035}]$	$({0}{\text{.035,0}}{.05}]$	$({0}{\text{.05,0}}{.08}]$
Method 1–2	3.1130E−04	4.0668E−04	4.745E−04	8.2437E−04
Method 2–2	3.1472E−04	4.1347E−04	4.8285E−04	8.0944E−04
Method 3	3.3844E−04	4.4702E−04	6.4585E−04	0.0012
Method 4	3.1202E−04	3.9477E−04	4.9058E−04	8.2475E−04
The proposed Method	1.8852E−04	9.4439E−05	2.3281E−04	0.0010

As could be seen from Table 2, in most areas, the RMSE calculate by the proposed method for different heights is the smallest. Compared with method 1–2, the accuracy of the proposed method is improved by 39.44%. Compared with method 2–2, the accuracy of the proposed method is improved by 40.10%. Compared with method 3, the accuracy of the proposed method is improved by 44.30%. Compared with method 4, the accuracy of the proposed method is improved by 39.58%. From the above analysis, it could be concluded that the proposed method could achieve higher-quality phase-to-height mapping with fewer mapping equations.

## Conclusion

In this paper, an improved phase-to-height mapping method combine with device attitude is proposed. Firstly, it is concluded that the unwrapped phase will be offset due to the influence of the device attitude in the PMP system. To solve this problem, this method uses GRNN to eliminate the phase offset. Finally, the unwrapped phase of eliminating the phase offset is substituted into the phase-to-height mapping equation, and the phase information is mapped to the height information with high quality. In order to verify the proposed method, a comparison experiment is carried out between the proposed method and the comparison method. Experimental results show that the proposed method has higher measurement accuracy with using less memory space. Compared with direct phase-to-height mapping method and nonlinear phase-to-height mapping method, the proposed method greatly reduces the memory space of mapping equations. Compared with the phase-to-height mapping method using PNN to eliminate phase offset, the measurement accuracy of the proposed method is improved by 44.30%. Compared with the phase-to-height mapping method of using RBF neural network to eliminate phase offset, the measurement accuracy of proposed method is improved by 39.58%. However, this paper do not further analyze the phase offset and its related factors, only use the phase offset at rated height as input to the GRNN. This still some phase error in the result. In future research, we will explore this issue. The proposed method provides a new idea and theoretical basis for the application of phase-to-height mapping method.
